# Mimicking of Estradiol Binding by Flame Retardants and Their Metabolites: A Crystallographic Analysis

**DOI:** 10.1289/ehp.1306902

**Published:** 2013-08-19

**Authors:** Rajendrakumar A. Gosavi, Gabriel A. Knudsen, Linda S. Birnbaum, Lars C. Pedersen

**Affiliations:** 1 Laboratory of Structural Biology, National Institute of Environmental Health Sciences, and; 2 Laboratory of Toxicology and Toxicokinetics, National Cancer Institute, National Institutes of Health, Department of Health and Human Services, Research Triangle Park, North Carolina, USA

## Abstract

Background: Brominated flame retardants (BFRs), used in many types of consumer goods, are being studied because of concerns about possible health effects related to endocrine disruption, immunotoxicity, reproductive toxicity, and neurotoxicity. Tetrabromobisphenol A (TBBPA), the most widely used BFR, and human metabolites of certain congeners of polybrominated diphenyl ether (e.g., 3-OH-BDE-47) have been suggested to inhibit estrogen sulfotransferase, potentially affecting estrogen metabolism.

Objectives: Our primary goal was to understand the structural mechanism for inhibition of the hormone-metabolizing enzyme estrogen sulfotransferase by certain BFRs. We also sought to understand various factors that facilitate the binding of flame retardants in the enzyme binding pocket.

Methods: We used X-ray crystallography to obtain atomic detail of the binding modes of TBBPA and 3-OH-BDE-47 to estrogen sulfotransferase for comparison with binding of the endogenous substrate estradiol.

Results: The crystal structures reveal how BFRs mimic estradiol binding as well as the various interactions between the compounds and protein residues that facilitate its binding. In addition, the structures provide insights into the ability of the sulfotransferase substrate binding pocket to accommodate a range of halogenated compounds that satisfy minimal structural criteria.

Conclusions: Our results show how BFRs or their metabolites can bind to and inhibit a key hormone-metabolizing enzyme, potentially causing endocrine disruption.

Citation: Gosavi RA, Knudsen GA, Birnbaum LS, Pedersen LC. 2013. Mimicking of estradiol binding by flame retardants and their metabolites: a crystallographic analysis. Environ Health Perspect 121:1194–1199; http://dx.doi.org/10.1289/ehp.1306902

## Introduction

Properties of brominated flame retardants (BFRs) that reduce flame propagation during fires have resulted in increased utilization of BFRs in electronic devices, building materials, furniture, automobiles, and airplanes (Green 1996; Shaw et al. 2010). BFRs are primarily categorized as additive or reactive (Alaee et al. 2003). Additive BFRs, such as polybrominated diphenyl ethers (PBDEs), are used in polyurethane foam, textiles, and a wide array of polymer-based products, where they are simply blended into the polymers and therefore can easily leach out of the products (Alaee et al. 2003). PBDEs were produced as mixtures of bromination content and called penta-, octa-, and deca-BDEs, with one of the major congeners in the penta-BDE mix being 2,2´,4,4´-tetrabromodiphenyl ether (BDE-47) (Alaee et al. 2003; Birnbaum and Staskal 2004). In contrast, reactive BFRs are chemically bonded into the plastics and heavily used in printed circuit boards, containing up to 20% bromine (Alaee et al. 2003). TBBPA (3,3´,5,5´-tetrabromobisphenol A), the most heavily produced BFR (with a worldwide demand of > 200,000 tons/year), is mainly used as a reactive BFR, but it has additional applications as an additive in products such as acrylonitrile–butadiene–styrene plastic products [Alaee et al. 2003; Birnbaum and Staskal 2004; Bromine Science and Environment Forum (BSEF) 2012; Environment Canada/Health Canada 2012].

A primary concern in the use of BFRs, such as PBDEs and TBBPA, is the large number of studies showing environmental release of these compounds from existing or discarded products (de Wit 2002; Stapleton et al. 2012b). These chemicals have been detected in air samples and sewage and river sediments (de Wit 2002). Several studies have reported nanograms per gram levels of these chemicals in breast milk and serum (Abdallah and Harrad 2011; Thomsen et al. 2002). A study of 77 children 1–3 years of age emphasized exposure of toddlers to BFRs in the home (Stapleton et al. 2012a). Exposure to BDE-47 and TBBPA has been associated with disruption in calcium signaling, immune response, and neurotoxicity (Koike et al. 2012; Mariussen and Fonnum 2003; Ogunbayo et al. 2008). TBBPA has been shown to induce tumor formation in rats and mice in a 2-year bioassay study conducted by the National Toxicology Program (2013). BFRs are structurally similar to hormones, and *in vitro* evidence has suggested that BFRs may mimic hormones and interfere with their binding, transport, and regulation (Chan and Chan 2012; Hamers et al. 2006), leading to endocrine disruption. Hamers et al. (2006) reported dose–response relationships of BFRs for interference with androgenic, estrogenic, and progesteronic pathways. Furthermore, crystal structures of PPARγ (peroxisome proliferator-activated receptor γ) in complex with TBBPA and tetrachlorobisphenol A (TCBPA) suggest how brominated and chlorinated flame retardants can mimic binding of ligands to receptors (Riu et al. 2011a).

To further complicate matters, BFRs may be able to act synergistically as endocrine disruptors, as suggested in a study involving BDE-47 and BDE-99 (Tagliaferri et al. 2010). The role of BFRs as endocrine disruptors might be further pronounced because of contribution of their metabolites/analogs, which have been previously detected in various species (Fini et al. 2012; Hakk et al. 2000; Schauer et al. 2006; Shen et al. 2012; Zalko et al. 2006). Hydroxylated metabolites of BDE-47 have been detected in incubations with rat microsomes (Hamers et al. 2008) and human hepatocytes (Marteau et al. 2012), as well as in fetal and maternal blood samples (Qiu et al. 2009). One of the metabolites detected by Hamers et al. (2008) and Qiu et al. (2009) was 3-hydroxy-2,2´,4,4´-tetrabromodiphenyl ether (3-OH-BDE-47). Some proteins may bind to metabolites but show negligible binding to the parent compound (Hamers et al. 2008).

In humans, there are 13 cytosolic sulfotransferases that catalyze the transfer of a sulfuryl group (SO_3_) from the donor cofactor 3´-phosphate 5´-phosphosulfate (PAPS) to acceptor substrates, including xenobiotics, fatty acids, neurotransmitters, and steroids (Gamage et al. 2006; Song 2001; Wang and James 2006). Sulfation of 17β-estradiol (E2) by human estrogen sulfotransferase (SULT1E1) results in loss of binding to the estrogen receptor as well as increased availability for renal excretion, thereby effectively regulating the concentration of E2 (Falany 1997). TBBPA and 3-OH-BDE-47 have been reported to inhibit SULT1E1 with IC_50_ (median inhibitory concentration) values of 33 and 23 nM, respectively (Hamers et al. 2008; Kester et al. 2002). Increased levels of penta-BDEs (BDE-47, BDE-99, and BDE-100) in house dust have been positively associated with increased serum E2 concentrations in 62 men recruited from an infertility clinic (Johnson et al. 2013). Together, these reports suggest that exposure to BFRs may affect the concentration of E2 in the cell, potentially triggering downstream estrogenic responses.

In the present study, we obtained crystal structures of SULT1E1 with the product cofactor adenosine-3´-5´-diphosphate (PAP) in complexes with the ubiquitous flame retardant TBBPA and a BFR metabolite, 3-OH-BDE-47. We compared these structures with that of E2 binding to understand in atomic detail how these compounds are accommodated and how they inhibit the hormone-metabolizing enzyme, SULT1E1.

## Materials and Methods

**Figure 1:**
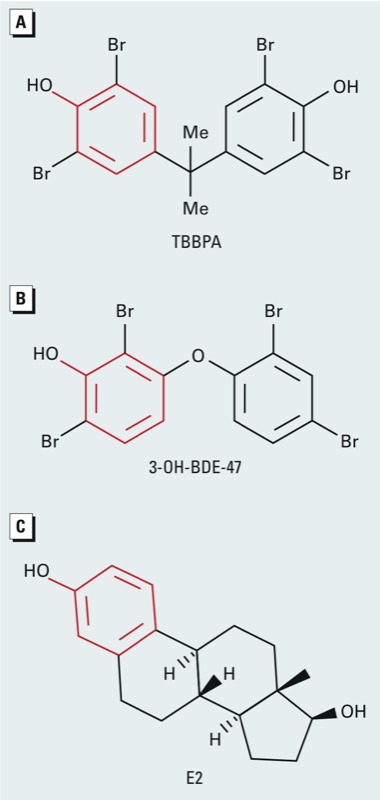
Chemical structures of (*A*) TBBPA, (*B*) 3‑OH-BDE‑47 (3-hydroxylated metabolite of the parent BFR, BDE‑47), and (*C*) 17β-estradiol (E2). Me, methyl group. The phenolic ring that the three compounds have in common is indicated in red.

*Chemicals.* TBBPA (analytical grade; Figure 1A), E2 (Figure 1C), and PAP, all ≥ 97% purity, were purchased from Sigma-Aldrich (St. Louis, MO); and 3-OH-BDE-47 (97% purity; Figure 1B) was purchased from AccuStandard (New Haven, CT).

*Protein expression, purification, and crystallization.* In these experiments, we used the SULT1E1 mutant V269E [expressed and purified as described previously (Pedersen et al. 2002)] because it facilitates crystallization. This mutation lies on the surface of the protein remote from the active site at the dimer interface and favors monomer formation in solution, yet it still forms the expected physiological dimer in the crystal, as seen in other sulfotransferase structures (Pedersen et al. 2002; Petrotchenko et al. 2001). Crystal structures of the complex of SULT1E1–PAP with TBBPA and with E2 were obtained by co-crystallization studies. The protein used for crystallization was concentrated to 13.6 mg/​mL in a solution at pH 7.5 and containing 1.5 mM sodium phosphate dibasic, 0.15 mM monopotassium phosphate, 40 mM sodium chloride, 1 mM dithiothreitol, and 4 mM PAP. TBBPA or E2 dissolved in 100% dimethyl sulfoxide (DMSO) was added to the protein stock for a final concentration of 8 mM. Protein–TBBPA solution or protein–E2 solution was mixed in equal volume with 0.1 M HEPES, pH 7.5, and 18–24% (wt/vol) polyethylene glycol 8000. For crystal growth, we used sitting drop vapor diffusion at 293K. Crystals were transferred to a cryoprotectant solution containing 0.1 M HEPES, pH 7.5, 22% polyethylene glycol 8000, 4 mM PAP, 15% ethylene glycol, and 8 mM TBBPA or E2 and flash frozen in liquid nitrogen.

To obtain the crystal structure of SULT1E1 in complex with 3-OH-BDE-47 and PAP, protein stock was mixed in equal volume with 0.1 M 2-[*N*-morpholino]​ethane sulfonic acid, pH 6.0, and 17–22% (wt/vol) polyethylene glycol 8000, then placed at 293K, where crystals were grown using hanging drop vapor diffusion. SULT1E1–PAP crystals were transferred in three steps into a cryoprotectant solution consisting of 3-OH-BDE-47 suspended at a concentration of 5 mM in 0.1 M 2-[*N*-morpholino]ethane sulfonic acid, pH 6.0, 20% (wt/vol) polyethylene glycol 8000, 4 mM PAP, and 15% (vol/vol) ethylene glycol. Crystals were soaked in the cryoprotectant solution for 5 days before flash freezing in liquid nitrogen for data collection.

*Competitive crystallization experiment.* Crystal structure of the complex of SULT1E1–​PAP–E2–TBBPA was obtained by co-crystallization studies. TBBPA and E2 dissolved in 100% DMSO were added to the protein stock for final concentrations of 8 mM each. Protein–E2–TBBPA solution was mixed in equal volume with 0.1 M HEPES, pH 7.5, and 18–24% (wt/vol) polyethylene glycol 8000. Crystal were grown using sitting drop vapor diffusion at 293K. For data collections, crystals were then transferred to a cryoprotectant solution containing 0.1 M HEPES, pH 7.5, 22% (wt/vol) polyethylene glycol 8000, 4 mM PAP, 15% (vol/vol) ethylene glycol, 8 mM TBBPA, and 8 mM E2 and flash frozen in liquid nitrogen.

*Crystallographic data collection, processing, and structure refinement.* Data were collected for all the crystals using a Saturn 92 X-ray detector with a Micromax-007 HF X-ray generator (Rigaku, The Woodlands, TX). The crystallographic data statistics are presented in Supplemental Material, Table S1. All data were indexed and scaled using HKL-2000 data processing software (Otwinowski and Minor 1997). The structures were solved using the structure of SULT1E1 [Protein Data Bank (PDB; http://www.rcsb.org/pdb/home/home.do) ID 1G3M (Shevtsov et al. 2003)] as a starting model. Reference R_free_ reflections were maintained in all three structures. PHENIX, version 1.8 (Adams et al. 2010) and Coot, version 0.6.1 (Emsley et al. 2010) were used to obtain the structures by iterative cycles of refinement and model building. We assessed model quality using MolProbity, version 1.5.0.2 (Chen et al. 2010). All structural figures were prepared using PyMOL (http://www.pymol.org/).

Atomic coordinates and structure factors for the reported crystal structures have been deposited with the Protein Data Bank under PDB IDs 4JVM for the SULT1E1–PAP–TBBPA structure, 4JVN for the SULT1E1–PAP–3-OH-BDE-47 structure, and 4JVL for the SULT1E1–PAP–E2 structure.

## Results

To understand the binding and inhibition of estrogen sulfotransferase by BFRs, we obtained crystal structures of SULT1E1 in complex with the product cofactor PAP and three different compounds bound at the active site: the natural substrate (E2), a BFR (TBBPA), and a human BFR metabolite (3-OH-BDE-47) at resolutions of 1.95 Å, 2.0 Å, and 2.05 Å, respectively. SULT1E1 crystallizes with two molecules in the asymmetric unit (designated molecule A and molecule B) representing the proposed physiological dimer (Petrotchenko et al. 2001).

*Crystal structure of the SULT1E1–PAP–E2 complex.* The crystal structure of human SULT1E1 with PAP and E2 bound to the active site (see Supplemental Material, Figures S1A,B and S2A) is similar in overall fold and substrate binding as previously determined for the mouse estrogen sulfotransferase (Kakuta et al. 1997). In brief, E2 binds to a mostly buried hydrophobic pocket, with the sulfuryl acceptor hydroxyl of E2 within hydrogen bonding distance to the proposed catalytic base His107 and Lys105, placing it in proper position for catalysis (Kakuta et al. 1997; Pedersen et al. 2002). Also contributing to the positioning are Phe80 and Phe141, which flank the planar faces of the phenolic ring of E2. Phe80 and Phe141 have been suggested to function as a steric gate conferring substrate specificity for the enzyme (Petrotchenko et al. 1999) (see Supplemental Material, Figure S1C).

**Figure 2:**
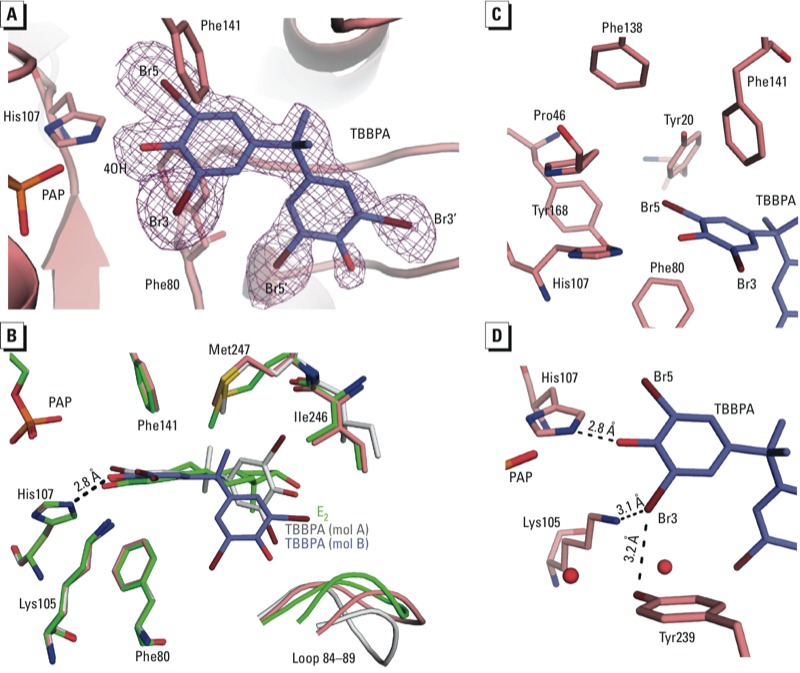
Crystal structure complex of SULT1E1–PAP–TBBPA. (*A*) A simulated annealing F_o_ – F_c_ omit map (purple) contoured at 2.5σ for TBBPA (blue), with bromine atoms shown in brown. SULT1E1 is shown in cartoon representation with His107, Phe80, and Phe141 colored salmon. (*B*) Superimposition of TBBPA-bound structure in molecule (mol) B (protein in salmon, TBBPA in blue) with E2-bound structure in green (mol B) [root mean square deviation (RMSD) of 0.09 Å over 233 Cα atoms], and TBBPA-bound structure in mol A (gray) with E2-bound structure in green (mol B) (RMSD of 0.171 Å over 225 Cα atoms). The phenolic hydroxyl of TBBPA (mol B) is within hydrogen bonding distance to the catalytic His107. There are no significant differences between the two structures except for loop residues 84–89. (*C–D*) Binding sites for Br5 (*C*) and Br3 (*D*).

*Crystal structure of the SULT1E1–PAP–TBBPA complex.* The co-crystal structure of PAP and TBBPA bound to SULT1E1 reveals TBBPA binding in the same substrate binding pocket as E2. In molecule B, the position of the TBBPA is well determined because there is clear electron density for the entire molecule, with strong density for the first phenolic ring and slightly weaker density for the second ring, suggesting that this ring contains greater conformational flexibility (Figure 2A). The first phenolic ring of TBBPA superimposes with the phenolic ring of E2, positioning the 4-hydroxyl within hydrogen bonding distance to the catalytic base His107, similar to the 3-hydroxyl of E2 (Figure 2B; see also Supplemental Material, Figure S2B). In addition, this ring neatly fits between the steric gate residues Phe80 and Phe141. The second ring is located out of the plane with respect to E2 and extends into a different region of the hydrophobic binding pocket (Figure 2B). The halogens on the first phenolic ring are easily accommodated within the active site of SULT1E1. The bromine (Br5) bound to the C5 carbon is buried in a hydrophobic cavity within van der Waal distance (4.3 Å) of Tyr20, Phe80, His107, Phe141, and Tyr168 (Figure 2C). Bromine atom Br3 is located in a more hydrophilic environment and is potentially able to form hydrogen or halogen bonds with Lys105 (3.1 Å) and/or Tyr239 (3.2 Å) (Figure 2D). On the second phenolic ring, Br3´ is located proximal to Leu88, Ile246, and Leu242 near the surface of the protein (see Supplemental Material, Figure S3A), whereas bromine Br5´ is surrounded by residues Phe80, Met89, and Phe75 and is also within hydrogen or halogen bonding distance to two ordered water molecules (3.3 Å and 3.2 Å) (see Supplemental Material, Figure S3B).

Overall, the protein conformation of SULT1E1 with bound TBBPA is very similar to that with bound E2 with only a few minor changes in the substrate binding pocket. Binding of TBBPA results in increased sidechain order for Met247, whereas the sidechain of Ile246 becomes slightly more disordered and the conformation of loop residues 84–89 is altered (2.0 Å shift at Cα atom of Asn87 in molecule B) (Figure 2B). Although the electron density for TBBPA in molecule A is not as clear for the second ring, it appears that the overall position of the molecule is slightly shifted with respect to the position in molecule B. This shift is correlated with a shift in the loop (4.5 Å at Cα atom of Asn87 in molecule A compared with molecule B of the E2-bound structure) containing residues 84–89.

**Figure 3:**
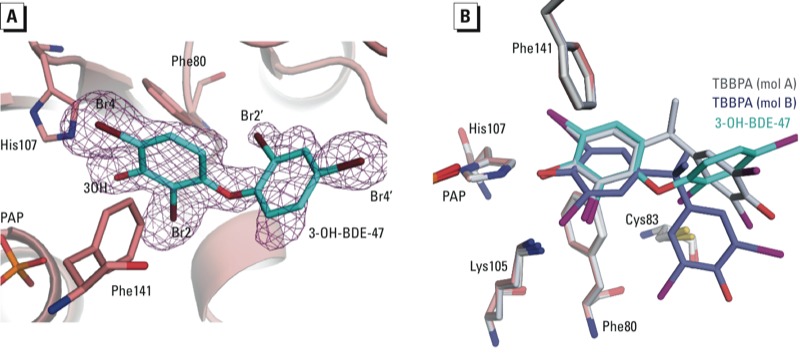
Crystal structure complex of SULT1E1-PAP-3-OH-BDE-47. (*A*) A simulated annealing F_o_ – F_c_ omit map (purple) contoured at 2.5σ for 3-OH-BDE-47 (cyan) with bromine atoms shown in brown. SULT1E1 is shown in cartoon representation with His107, Phe80, and Phe141 colored salmon. (*B*) Superimposition of SULT1E1 structure with bound 3-OH-BDE-47 to that of SULT1E1 with bound TBBPA [gray: mol A (RMSD = 0.1 over 257 Cα atoms); blue; mol B (RMSD = 0.21 over 241 Cα atoms)]. The first phenolic rings of each compound superimpose well despite different orientation of the second phenolic ring.

*Crystal structure of the SULT1E1–PAP–3-OH-BDE-47 complex.* The crystal structure of SULT1E1 with PAP and the BDE-47 metabolite 3-OH-BDE-47 bound reveals that the metabolite is bound to the same binding site as E2 and TBBPA (Figure 3; see also Supplemental Material, Figure S2C). Similar to TBBPA, there is strong electron density for the phenolic ring of 3-OH-BDE-47 bound to both molecules A and B but much weaker density for the second aromatic ring, which is modeled as partial occupancy in molecule A and is too weak to model in molecule B (Figure 3A). The phenolic ring superimposes well with that from TBBPA in both molecules A and B, forming similar interactions with the protein (Figure 3B; see also Supplemental Material, Figure S3C,D). Compared with TBBPA, 3-OH-BDE-47 shows different substitution patterns on the phenolic ring (3-OH vs. 4-OH) and different bridging groups between the aromatic rings (ether vs. isopropyl) creating divergence in the positioning of the second aromatic ring (Figure 3B). For 3-OH-BDE-47, the dibromophenyl group is overlapping with, but perpendicular to, the plane of the E2 molecule in the E2 bound structure (see Supplemental Material, Figure S4D). This positions Br4´ at the protein surface exposed to the solvent (Figure 3A). The density for Br2´ is weaker than that for Br4´, likely due to different rotamers of the second ring, which would maintain the position of Br4´ but reposition Br2´ in multiple orientations. Binding of 3-OH-BDE-47 in molecule A results in a shift in the position of loop 84–89 (4.1 Å shift at Cα on Asn87) compared with E2 binding, similar to what is seen in molecule A when TBBPA is bound.

**Figure 4:**
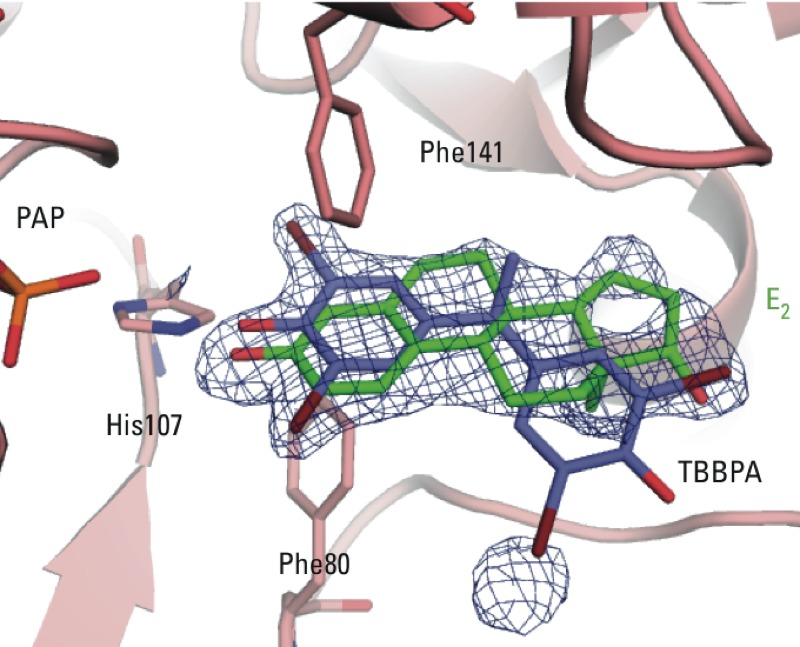
Crystal structure complex of SULT1E1–PAP–E2–TBBPA. Unmodeled F_o_ – F_c_ electron density (purple) contoured at 2.5σ shows partial density for both E2 (green) and TBBPA (blue). E2 and TBBPA have been placed into the active site based on the superimpositions of their respective complexes with SULT1E1.

*Co-crystallization of PAP, TBBPA, and E2 to SULT1E1.* To examine whether TBBPA could compete for binding with E2, we co-crystallized SULT1E1 in the presence of PAP and equal concentrations of TBBPA and E2. Unmodeled electron density contoured at 2.5σ in the substrate binding site suggests that E2 and TBBPA are competing for the same binding site (Figure 4). Strong electron density exists for the common phenol ring of the two molecules, but only partial density exists at the position where halogens are found on the first ring of TBBPA in molecule A of the TBBPA structure. There is also partial density for the remainder of the E2 molecule. This suggests that a certain percentage of SULT1E1 molecules in the crystal are binding E2, while others are binding TBBPA in the substrate binding site.

## Discussion

*Binding to sulfotransferase by BFRs.* In the present study we obtained crystal structures of SULT1E1 in complex with TBBPA (a BFR) and 3-OH-BDE-47 (a metabolite of a BFR, BDE-47). Although these chemicals differ structurally, there are remarkable similarities in how they bind to SULT1E1. A noticeable structural feature is the presence of a phenolic ring with the hydroxyl flanked by two bromine atoms (Figure 1, in red). The phenolic ring on these BFRs superimposes well with that of the acceptor phenol of E2, and is selected for by steric gate residues Phe80 and Phe141, which have been reported to contribute toward substrate specificity (Petrotchenko et al. 1999). The absence of the hydroxyl on BDE-47 increases the IC_50_ value to SULT1E1 by 170-fold compared with that of the metabolite 3-OH-BDE-47 (Hamers et al. 2008). This suggests that the hydroxyl moiety on the BFRs enhances the binding affinity to SULT1E1.

In addition to the hydroxyl moiety, bromine atoms appear to contribute to stable binding of BFRs. Bromine atoms substituted on adjacent carbons to the hydroxyl are not only tolerated in SULT1E1 but appear to enhance binding affinities, as observed for other halogens in different systems (Gales et al. 2008; Riu et al. 2011a). This is supported by the fact that bisphenol A (BPA), the nonbrominated form of TBBPA, has an IC_50_ value for SULT1E1 300 times higher than that of TBBPA (Kester et al. 2002). Consistent with the poor inhibition by BPA, our attempts to crystallize SULT1E1 in complex with PAP and BPA resulted in no detectable binding for BPA (data not shown). The ability of some cytosolic sulfotransferases such as SULT1E1 to have enhanced binding to halogenated compounds may stem from physiological roles in sulfation of iodothyronines. Thyroid hormones have iodine atoms substituted on the phenolic ring adjacent to the acceptor hydroxyls and have been shown to be substrates for SULT1E1 (Kester et al. 1999). Sulfation is an important step in the inactivation and metabolism of these hormones (Visser et al. 1990).

Another factor allowing for inhibition of SULT1E1 by BFRs is the expanse of the substrate binding pocket away from the catalytic site. Neither TBBPA nor 3-OH-BDE-47 are planar, like E2, resulting in the second aromatic ring being out of the plane compared with the position of E2 (see Supplemental Material, Figure S4A–D). The size of the pocket allows for binding of various ligands at this position in various orientations (see Supplemental Material, Figure S5). Compared with the first ring, the second aromatic ring in TBBPA and 3-OH-BDE-47 likely do not contribute significantly to their affinity for binding. This is exemplified by the decrease in the electron density in this region of both molecules that is consistent with increased disorder (Figures 2A and 3A). The IC_50_ values of TBBPA and 3-OH-BDE-47 (33 and 23 nM, respectively) are near the *K*_m_ of 5 nM for E2, suggesting that these compounds bind to SULT1E1 with high affinity (Hamers et al. 2008; Kester et al. 2002; Zhang et al. 1998). It is possible that the bromine atoms on the first ring of the compound may compensate for the lack of specific interactions at the disordered end, compared with the rigid E2 molecule. Another example of SULT1E1 accommodating a halogenated aromatic hydrocarbon is a previously reported structure of SULT1E1 in complex with PAP and 4,4´-(OH)_2_-3,5,3´,5´-tetrachlorobiphenyl [a hydroxylated form of PCB (polychlorinated biphenyl)-80] (Shevtsov et al. 2003). Based on superimpositions of the SULT1E1 structures bound to TBBPA, 3-OH-BDE-47, 4,4´-(OH)_2_-3,5,3´,5´-tetrachlorobiphenyl, and E2, it appears that SULT1E1 is able to bind these structurally diverse compounds with only minor changes in a few sidechain residues and small shifts in loop 84–89 (see Supplemental Material, Figures S5 and S6). The ability to accommodate structurally diverse compounds provides an opportunity for the enzyme to bind a large variety of halogenated phenols, suggesting that inhibition of SULT1E1 could occur at lower individual doses when exposed to mixtures of BFRs. Despite the seemingly promiscuous nature of the substrate binding pocket to polyhalogenated aromatic phenols, not all are capable of potent inhibition of SULT1E1 (Hamers et al. 2008; Kester et al. 2000, 2002). The metabolites 6-OH-BDE-47 and 2´-OH-BDE-66 have IC_50_ values for SULT1E1 that are approximately 20- and 80-fold higher than that of 3-OH-BDE-47. These compounds differ from TBBPA and 3-OH-BDE-47 because their hydroxyls are in *ortho* positions. Such an arrangement would likely not allow for the hydroxyl to sit at the “sulfuryl acceptor” position and thus would be unable to form hydrogen bonds with His107 and Lys10, resulting in decreased binding affinity.

*Inhibition of sulfotransferases by BFRs.* Previous kinetic studies on BFR inhibition of SULT1E1 suggest that the mode of inhibition is by a noncompetitive mechanism (Kester et al. 2002). This is consistent with work that demonstrated substrate inhibition via an allosteric binding site (Zhang et al. 1998). Structural evidence for a noncompetitive binding site comes from the crystal structure of the sulfotransferase SULT1A1 in complex with the substrate *p*-nitrophenol, with two molecules bound within the substrate binding pocket (Gamage et al. 2003). The crystal structures of E2 bound to estrogen sulfotransferases (mouse and human) do not reveal such an allosteric site (Kakuta et al. 1997). Our crystallization experiment in the presence of both E2 and TBBPA suggests that the binding of E2 does not permit the binding of TBBPA at another position (Figure 4). Rather, the observed electron density is consistent with both molecules competing for binding at the same catalytic binding site. Noncompetitive inhibition patterns can also be observed for active site binding inhibitors in two substrate systems when the inhibitor shows preferential binding for a different conformation of the enzyme than the acceptor substrate (Blat 2010). This suggests that inhibition of SULT1E1 by specific BFRs may be a result of high-affinity binding to the SULT1E1-PAP postcatalytic complex (as in our crystal structures). This is consistent with recent kinetic studies that suggest that noncompetitive substrate inhibition by high concentrations of E2 is through a dead-end complex where both PAP and E2 are bound (Sun and Leyh 2010).

*Sulfation of BFRs.* To complicate matters, BFRs containing phenol groups not only function as inhibitors but are also weak substrates for the sulfotransferases. Several studies have detected sulfated TBBPA in urine and blood samples of rats and humans, as well as in tadpole extracts (Fini et al. 2012; Hakk et al. 2000; Schauer et al. 2006). TBBPA and TCBPA (tetrachlorobisphenol A) have been shown by *in vitro* studies to be modestly sulfated by SULT1A1 and SULT1E1 (Kester et al. 2002). In a superimposition of the crystal structures of SULT1E1–PAP–TBBPA, SULT1E1–PAP–3-OH-BDE-47, and SULT1E1–PAP–E2 with that of the SULT1E1–PAPS complex [PDB ID code 1HY3, (Pedersen et al. 2002)], all the atoms required for the transfer of the sulfuryl group to the hydroxyls of TBBPA and 3-OH-BDE-47 are positioned for catalysis similar to that seen for E2 sulfation (see Supplemental Material, Figure S5). Sulfation of these compounds could decrease their toxicity by enhancing their solubility and renal excretion. In addition, sulfation may decrease the compound’s toxicity via disruption of binding, because *in vivo* studies of the sulfoconjugate of TBBPA demonstrate only the parent compound having an effect on thyroid hormone signaling (Fini et al. 2012). In contrast, studies on PPARγ suggest that sulfated TBBPA still exhibits residual PPARγ binding (Riu et al. 2011b). Because of uncertainty in the fate and consequences of the BFRs, their metabolites, or conjugates in the cell, further studies are necessary to understand their contribution and mechanism in endocrine disruption.

## Conclusion

BFRs may disrupt proper endocrine function by multiple mechanisms including hormone signaling, transport, and metabolism. Here we have presented crystal structures of the BFR TBBPA and a human BFR metabolite 3-OH-BDE-47 bound to the steroid-metabolizing enzyme SULT1E1. These structures reveal how BFRs can mimic estradiol binding to the active site of the enzyme. TBBPA and 3-OH-BDE-47 are structurally diverse from each other, as well as from estradiol, but can still be accommodated by the enzyme. The common feature is a phenolic ring. The presence of bromine atoms adjacent to the hydroxyl in some BFRs and their metabolites may compensate for the lack of similarity in structure to E2. Low IC_50_ inhibition by structurally diverse BFRs, including parent compounds and metabolites, suggests that low-dose exposure to multiple compounds could have an additive effect, reducing the concentration required of a single compound for endocrine disruption.

## Supplemental Material

(3.5 MB) PDF
