# Supplementary material for: Mimicking of Estradiol Binding by Flame Retardants and Their Metabolites: A Crystallographic Analysis
Source: Environ Health Perspect. 2013 Aug 19;121(10):1194–9. doi: 10.1289/ehp.1306902 (PMC3801471; doi:10.1289/ehp.1306902)
Supplement: (3.5 MB) PDF [file ehp.1306902.s001.pdf]

Environmental Health Perspectives doi:10.1289/ehp.1306902

Supplemental Material

Mimicking of Estradiol Binding by Flame Retardants and Their Metabolites: A Crystallographic Analysis

Rajendrakumar A. Gosavi, Gabriel A. Knudsen, Linda S. Birnbaum, and Lars C. Pedersen

Address correspondence to L. C. Pedersen, MD F3-09, 111 T W Alexander Dr., Research Triangle Park, NC 27709 USA. Phone: (919) 541-0444. Email: pederse2@niehs.nih.gov

Table of Contents:

Supplemental Material, Table S1, page 2: Crystallographic data statistics.

Supplemental Material, Figure S1, page 3: Crystal structure complex of SULT1E1-PAP-E2.

Supplemental Material, Figure S2, page 4: Ligand binding to SULT1E1.

Supplemental Material, Figure S3, page 5: Bromine atom binding sites for TBBPA and 3-OH-BDE-47.

Supplemental Material, Figure S4, page 6: Relative positions of BFR binding in SULT1E1.

Supplemental Material, Figure S5, page 7: Binding of structurally diverse ligands to the substrate binding pocket of SULT1E1.

Supplemental Material, Figure S6, page 8: RMSF (root mean square fluctuation) for residues that line the substrate binding pocket of SULT1E1.

References, page 9.

Supplemental Material, Table S1. Crystallographic data statistics.

**Parameters**	**SULT1E1-PAP-TBBPA**	**SULT1E1-PAP-3-OH-BDE-47**	**SULT1E1-PAP-E2**
PDB ID code	4JVM	4JVN	4JVL
Data Collection			
Unit cell dimensions (in Å)	a = 62.6, b = 97.4, c = 61.8	a = 62.7, b = 96.9, c = 61.4	a = 62.8, b = 96.9, c = 61.9
Space group	$P2_1$	$P2_1$	$P2_1$
No. of observations	303,922	260,032	198,916
Unique reflections	49,762	45,316	54,299
R_{sym} (%) (last shell)	11.9 (54.8)	13.8 (32.5)	8.4 (34.5)
I/σI (last shell)	13.2 (2.0)	9.3 (2.3)	14.3 (2.3)
Completeness (%) (last shell)	99.0 (93.7)	98.5 (89.5)	99.8 (99.9)
Refinement statistics			
Resolution (Å)	2.0	2.05	1.95
R_{cryst} (%)	18.3	18.5	17.3
R_{free} (%)[a]	23.0	23.5	21.6
Complexes in asymmetric unit	2	2	2
Root mean square deviation from ideal values			
Bond Length (Å)	0.008	0.004	0.007
Bond angle (degrees)	1.081	0.919	1.136
Ramachandran statistics			
Residues in favored regions	96.4	97.0	97
Residues in allowed regions	100	100	99.7

[a]Reference R_{free} reflections were maintained.

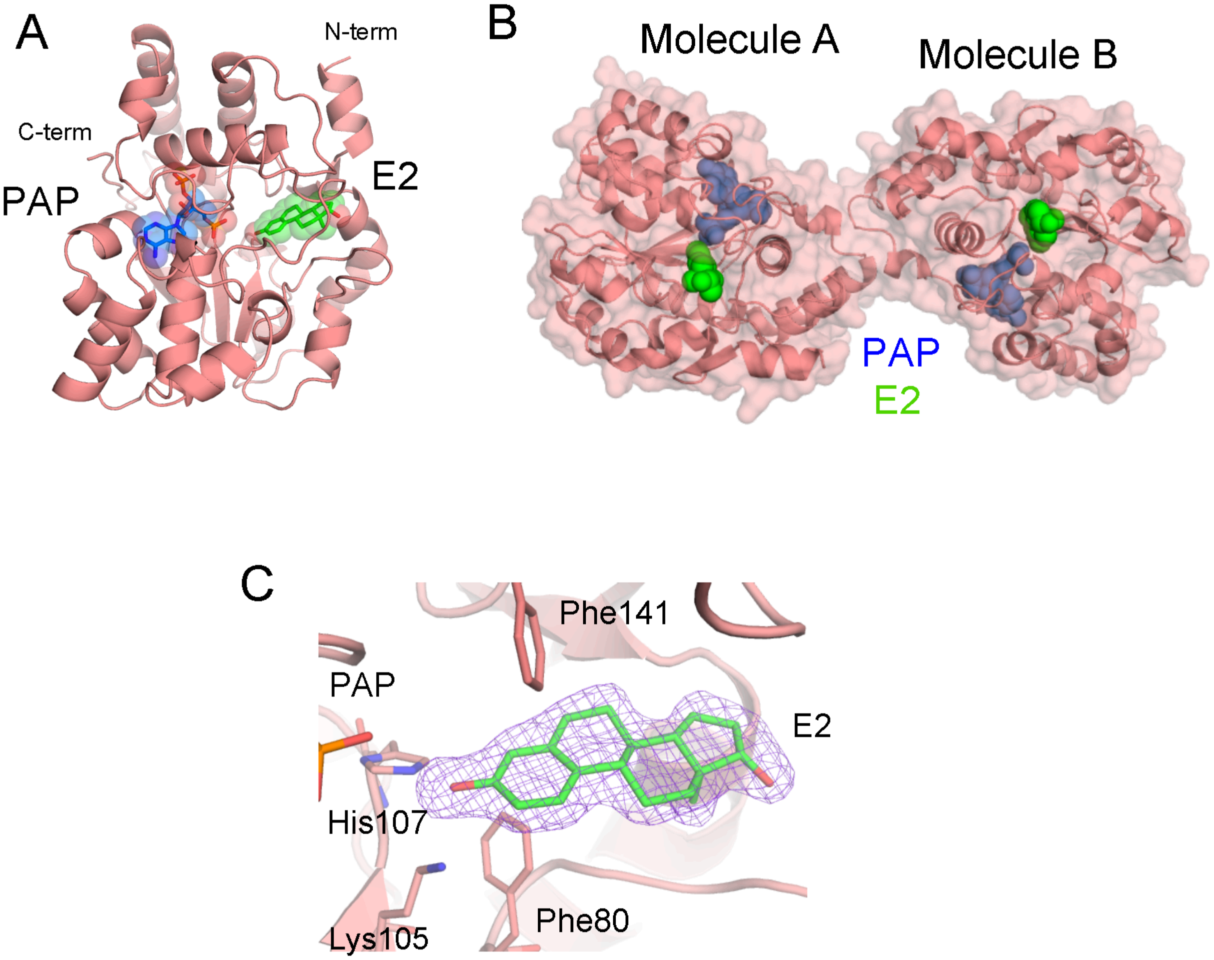

Supplemental Material, Figure S1. Crystal structure complex of SULT1E1-PAP-E2. A) Monomer of SULT1E1 showing PAP (blue) and E2 (green). B) Dimer of the complex showing molecule A and molecule B with PAP and E2 shown in spheres. C) A simulated annealing F_o-F_c omit map (purple) for E2 contoured at 2.5σ. SULT1E1 is shown with His107, Lys105 and steric gate residues Phe141 and Phe80 colored salmon.

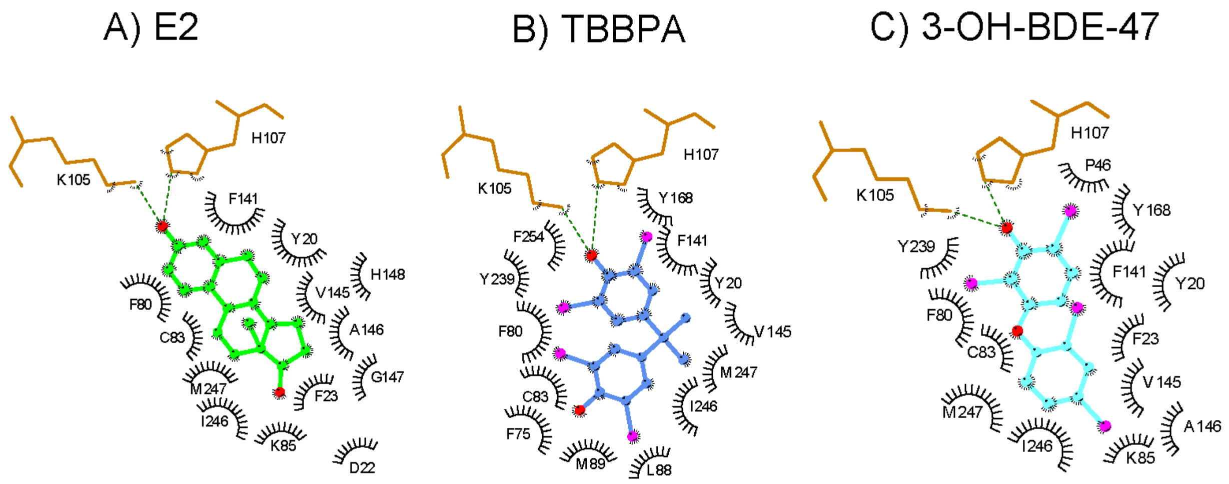

Supplemental Material, Figure S2. Ligand Binding to SULT1E1. Pictured are the residues that line the buried hydrophobic pocket of SULT1E1 based on a 4.3 Å cutoff for (A) E2, (B) TBBPA from molecule B and (C) 3-OH-BDE-47 from molecule A. Residues that form hydrogen bonds with the substrate are shown in orange and hydrogen bonds are displayed in green. Oxygen atoms on the ligands are displayed in red and bromine atoms are shown in magenta. The volume of the substrate binding pocket was calculated at 967 $Å^3$ with a total surface area of 787.8 $Å^2$. The amount of buried surface area of the binding pocket at the interface upon binding of E2, TBBPA, and 3-OH-BDE-47 is 366 $Å^2$, 419 $Å^2$, and 386 $Å^2$ respectively. Figures were created using LigPlus (Laskowski and Swindells 2011), buried surface area and pocket residues were determined in CNS (Brunger et al. 1998) and binding pocket calculations were carried out in CASTp (Dundas et al. 2006).

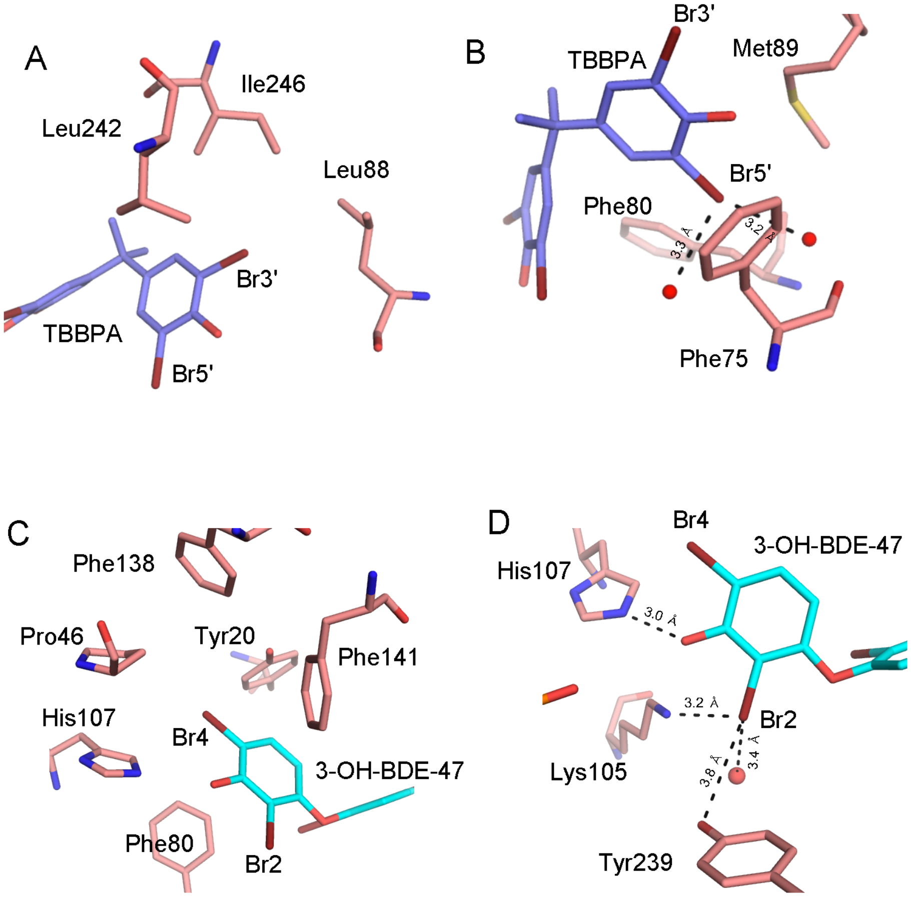

Supplemental Material, Figure S3. Bromine atom binding sites for TBBPA (panels A and B) and 3-OH-BDE-47 (panels C and D). TBBPA is shown in blue. 3-OH-BDE-47 is shown in cyan. Protein residues are shown in salmon. (A) and (B) Residues surrounding Br5' and Br3', respectively for TBBPA. (C) and (D) Residues surrounding Br4 and Br2, respectively for 3-OH-BDE-47.

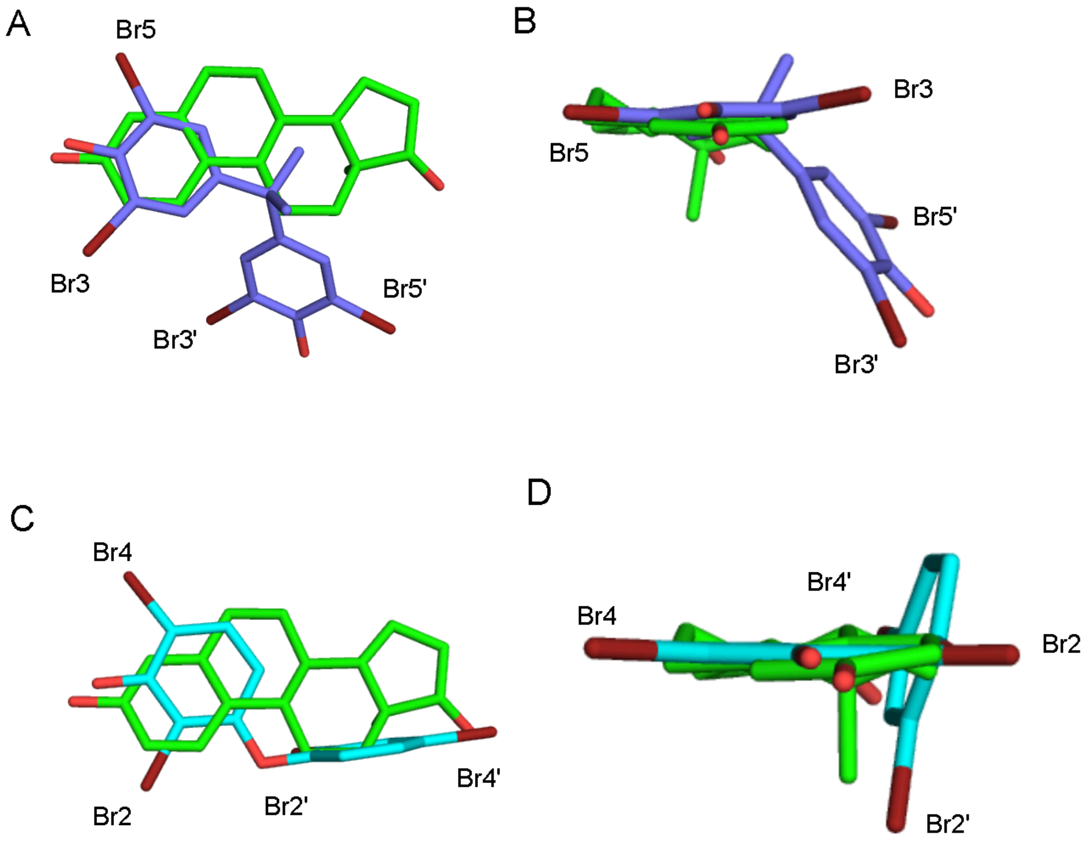

Supplemental Material, Figure S4. Relative positions of BFR binding in SULT1E1. Superposition of crystal structures of SULT1E1-PAP in complex with E2 (green), with TBBPA (blue) and 3-OH-BDE-47 (cyan). (A) Superposition of E2 and TBBPA in the active site of SULT1E1. (B) In-plane view showing the position of the second phenolic ring of TBBPA out of the plane of estradiol. (C) Superposition of E2 and 3-OH-BDE-47 in the active site of SULT1E1. (D) In-plane view showing the position of the second aromatic ring nearly perpendicular to the plane of estradiol. Bromine atoms on TBBPA and 3-OH-BDE-47 are labeled.

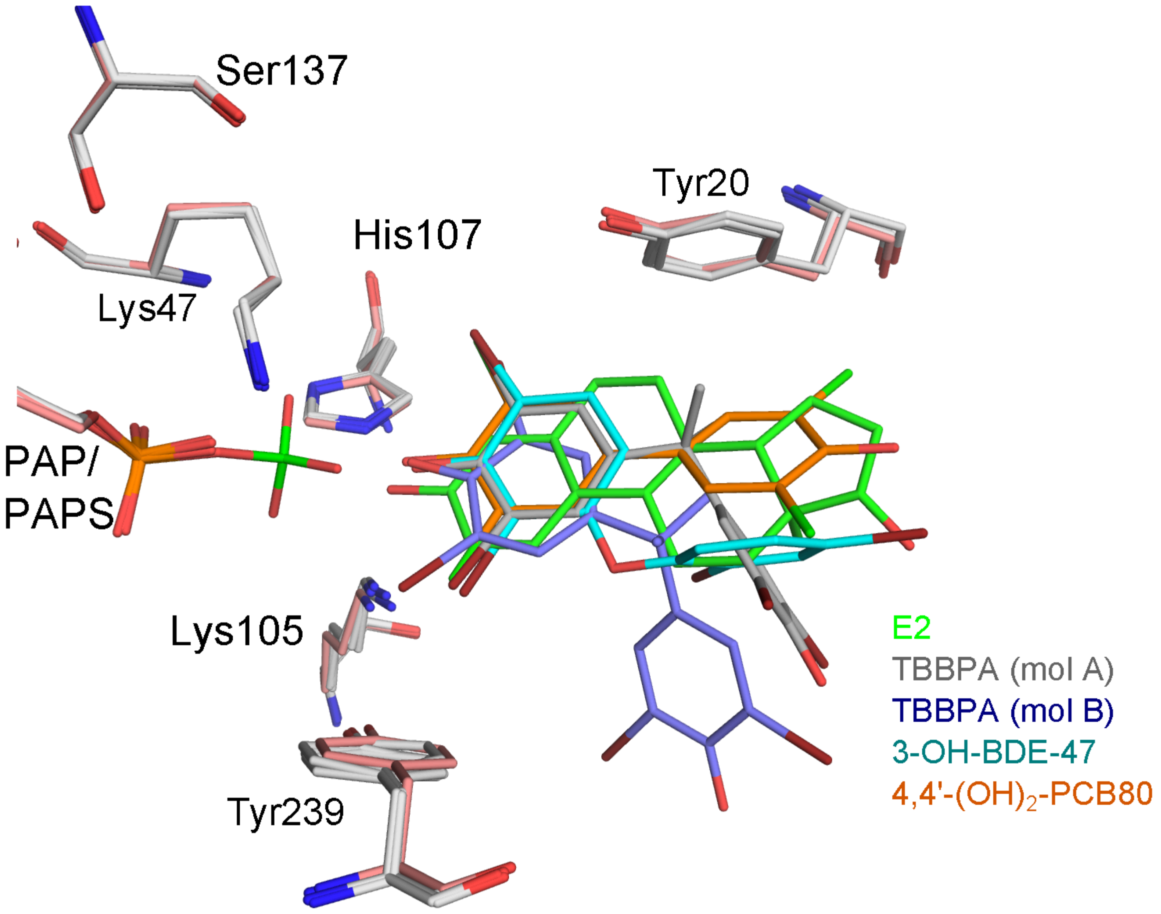

Supplemental Material, Figure S5. Binding of structurally diverse ligands to the SULT1E1 substrate binding pocket. Superimposition of crystal structures of SULT1E1 in complex with PAP and E2 (green), TBBPA (molecule A in grey and molecule B in blue), 3-OH-BDE-47 (cyan) and 4,4'-$(OH)_2$-3,5,3',5'- tetrachlorobiphenyl (a hydroxylated form of PCB80, PDB ID code 1G3M, (Shevtsov et. al. 2003)) (orange). A structure of SULT1E1 in complex with PAPS (PDB ID code 1HY3, (Pedersen et. al. 2002)) is also superimposed displaying the relative position of the donor group. Protein residues for E2 bound structure are shown in salmon and those for the other structures are shown in grey.

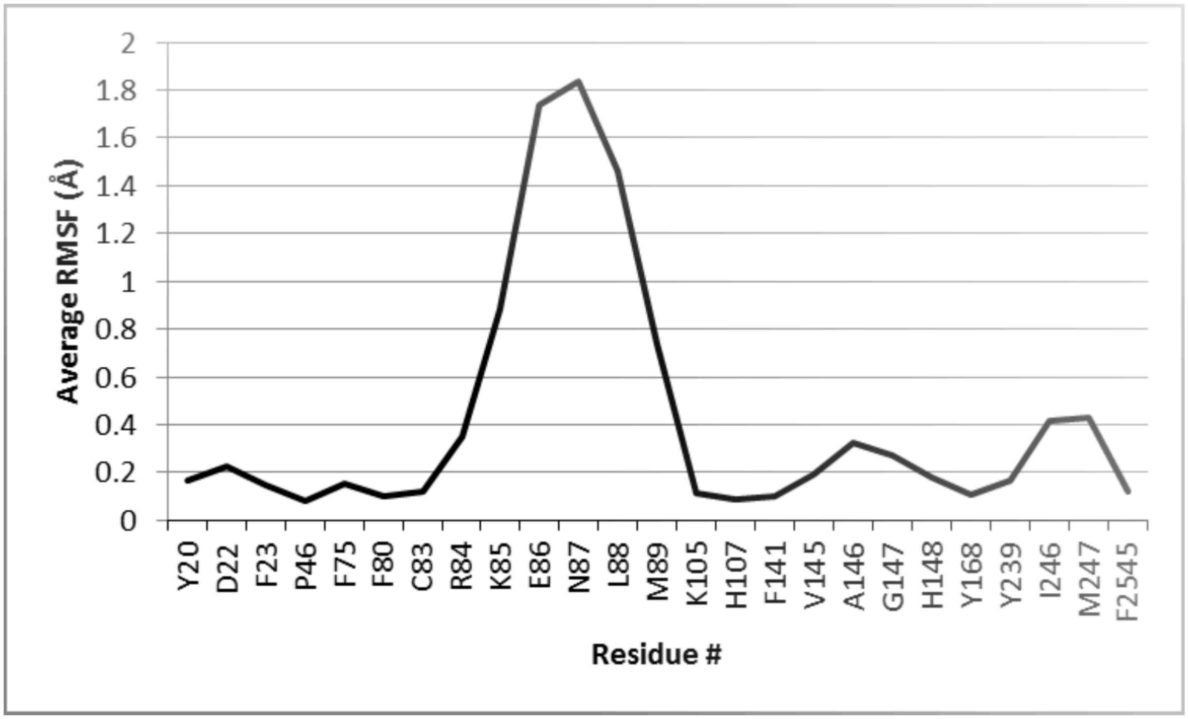

Supplemental Material, Figure S6. RMSF (root mean square fluctuation) for residues that line the substrate binding pocket of SULT1E1. Five structures of SULT1E1 with different ligands were evaluated: (1) SULT1E1 with E2 bound (molecule A), (2) SULT1E1 with 4,4'-$(OH)_2$-3,5,3',5'-tetrachlorobiphenyl bound (molecule A), (3) SULT1E1 with 3-OH-BDE-47 bound (molecule A), and (4 & 5) SULT1E1 with TBBPA bound (molecules A and B). To calculate the RMSFs, all structures (2-5) were superimposed onto molecule A of the SULT1E1 with E2 bound structure (RMSDs of 0.16 Å over 257 Cα atoms, 0.12 Å over 257 Cα atoms, 0.1 Å over 241 Cα atoms and 0.19 Å over 238 Cα atoms, respectively for 2-5). RMSF values for individual atoms across these structures within a given residue were averaged to get the average RMSF for a given residue. For residues 84-89, only backbone atoms were analyzed due to lack of clear density in all structures for the sidechains. With the exception of the loop containing residues 84-89 there is very little change upon the substrate binding with the different ligands.

References

Brunger AT, Adams PD, Clore GM, DeLano WL, Gros P, Grosse-Kunstleve RW, et al. 1998. Crystallography & nmr system: A new software suite for macromolecular structure determination. Acta Crystallogr D Biol Crystallogr 54:905-921.

Dundas J, Ouyang Z, Tseng J, Binkowski A, Turpaz Y, Liang J. 2006. Castp: Computed atlas of surface topography of proteins with structural and topographical mapping of functionally annotated residues. Nucleic Acids Research 34:W116-W118.

Laskowski RA, Swindells MB. 2011. Ligplot+: Multiple ligand-protein interaction diagrams for drug discovery. J Chem Inf Model 51:2778-2786.

Pedersen LC, Petrotchenko E, Shevtsov S, Negishi M. 2002. Crystal structure of the human estrogen sulfotransferase-paps complex - evidence for catalytic role of ser(137) in the sulfuryl transfer reaction. J Biol Chem 277:17928-17932.

Shevtsov S, Petrotchenko EV, Pedersen LC, Negishi M. 2003. Crystallographic analysis of a hydroxylated polychlorinated biphenyl (oh-pcb) bound to the catalytic estrogen binding site of human estrogen sulfotransferase. Environ Health Perspect 111:884-888.
